# The Reactions of Adolescents, Parents and Clinicians to Participating in Qualitative Research Interviews Regarding Adolescents Bereaved by Suicide and Other Traumatic Death

**DOI:** 10.3390/ijerph19010452

**Published:** 2022-01-01

**Authors:** Karl Andriessen, Karolina Krysinska, Debra Rickwood, Jane Pirkis

**Affiliations:** 1 Centre for Mental Health, Melbourne School of Population and Global Health, The University of Melbourne, Parkville, VIC 3010, Australia; karolina.krysinska@unimelb.edu.au (K.K.); j.pirkis@unimelb.edu.au (J.P.); 2 Faculty of Health, University of Canberra, Canberra, ACT 2617, Australia; Debra.Rickwood@canberra.edu.au

**Keywords:** grief, bereavement, suicide, traumatic death, research participation, ethics, research ethics

## Abstract

There are concerns that involving adolescents bereaved by suicide and other traumatic death in research may cause distress and harm. However, no study has investigated such bereaved adolescents’ research experiences. In addition, no study has looked at the experiences of parents and clinicians as participants in adolescent suicide and traumatic death bereavement research. This study aimed to explore the short-term impact of research participation experienced by adolescents, parents, and clinicians. A total of 61 participants (adolescents, *n* = 17; parents, *n* = 12; clinicians, *n* = 32) filled out a short survey within two weeks of having taken part in a qualitative interview study. Data were analyzed descriptively. Most participants had experienced no distress while participating and no negative effects of participating; rather, participation was experienced as helpful for them and they would highly recommend participating in a study like this to others. A few adolescents and parents reported some distress, related to anxiety about participation and the unpleasantness of grief memories. The study clearly indicates that bereaved adolescents, parents and clinicians can safely participate in research interviews regarding their experiences of grief and help after suicide, generally valuing the opportunity to share their experience. To prevent and mitigate potential distress, training of research staff and implementation of appropriate participant distress protocols are imperative. Future studies could include longitudinal follow-up of participants to assess any longer-term consequences.

## 1. Introduction

### 1.1. Rationale

Adolescents who are bereaved by the death of someone close to them, such as a family member or a friend, often experience short-term and long-term impacts regarding their grief, mental health and social functioning [1,2,3,4,5]. Experiencing a death is often an unfamiliar, disruptive and stressful event in their lives, leading to acute grief reactions such as crying, and feelings of numbness, sadness and longing for the deceased person [2,3,4,5]. Compared to other types of bereavement, adolescents bereaved by suicide and other traumatic death can experience more pronounced feelings of shock, guilt, anger, and abandonment [3,4,6]. They can struggle more with “why” questions, finding meaning in the loss, and experience less social support [1,2,3,6]. In addition, they have an increased risk of mental health problems, such as depression, anxiety, posttraumatic stress disorder, and long-term increased risk of suicidal behavior compared to other bereaved and non-bereaved adolescents [7,8,9].

While negative grief reactions are more prominent, there is also emerging evidence of personal or posttraumatic growth in this population [10,11]. The growth is understood as a positive psychological transformation that occurs as the result of a struggle with a traumatic and highly distressing event [12]. Traumatically bereaved adolescents can experience personal or posttraumatic growth in various domains, including increased appreciation of life and relationships, increased maturity and self-care, and finding new opportunities, for example, regarding school or professional career paths [3,11,12]. Nonetheless, bereavement by suicide and other traumatic death among adolescents can disconnect them from their friends and rupture the family equilibrium [13,14,15], which in turn may affect their parents’ and guardians’ capacity to support them and/or refer them to professional help [16].

Given the potential ramifications of bereavement by suicide and other traumatic death in adolescents, conducting research with this population poses important ethical challenges. The Australian National Statement on Ethical Conduct in Human Research (2007) (Updated 2018) [17] places value on principles of research merit and integrity, justice, beneficence, and respect. Researchers are required to minimize and manage potential risks to participants, and risks are only justified when they are outweighed by potential benefits for participants or the community. The National Statement [17] stipulates that research with bereaved minors/adolescents requires specific attention to participants’ capacity to understand the research and consent.

Important concerns regarding participant safety and the potential negative impact of research participation have been voiced in various research fields involving vulnerable populations, including suicide [18,19,20,21], trauma and violence [22,23], palliative care [24], psychiatry [25,26], and bereavement [27,28]. In studies with adults bereaved by suicide, research ethics committees have expressed concerns about potential harm to research participants, particularly that talking about grief experiences may traumatize them and increase their suicide risk [29,30,31]. Further, there are concerns about whether research participants who are negatively impacted will receive enough support [31].

Nonetheless, a recent systematic review found that most participants in suicide bereavement studies experience research participation positively [32]. Positive experiences included gaining insight into their grief experience and providing opportunities for helping others. However, a minority of participants reported unpleasant or negative experiences when participating in studies, such as being reminded of painful experiences, e.g., [27,33]. Despite the importance of these findings, the reviewed studies had several limitations [32]. Most were psychological autopsy studies (in which participants provide information about the deceased person rather than about themselves), and only one study included adolescents in the sample [34]. Hence, little is known about the experiences of adolescents of being involved in research regarding their own grief and help-seeking after a loss by suicide or other traumatic death. In addition, no study has looked at the experiences of parents and clinicians as participants in adolescent suicide and traumatic death bereavement research. This study aimed to address this gap by exploring the short-term impact of research participation experienced by adolescents, parents, and clinicians.

### 1.2. Background: Original Study

#### 1.2.1. Sampling

The original study, about which participants were then asked about their participation experience, was a qualitative study that examined how to best help adolescents bereaved by suicide and other traumatic death. Although details have been published [13,35], we summarize the original study here to provide context for the current study of participant experiences.

The original study adhered to the COREQ criteria [36] and involved a purposive sample of three groups of participants (adolescents, parents and clinicians) recruited in Australia between October 2019 and March 2020. Adolescents could participate if they had lost a family member or friend through suicide or other cause when they were aged between 12 and 18 years, and had experienced the death between six months and 10 years before participating in the study. Parents could participate if they were the parent of an eligible adolescent. Adolescents and parents could participate whether or not their parents or adolescent children participated. Clinicians could participate if they had at least five years of experience with providing professional help to bereaved adolescents.

Study participants (*N* = 72) included a total of 20 adolescents, 18 parents and 34 clinicians. The adolescent participants (16 girls, 4 boys) were aged 14 to 26 years (*M* = 19.50, *SD* = 2.95). They had lost their father (*n* = 9), brother (*n* = 3), sister (*n* = 2), mother (*n* = 2), other family member (*n* = 2), or friend (*n* = 2), by suicide (*n* = 18) or by accident (*n* = 2), on average 4 years previously (*M* = 3.92, *SD* = 2.49, range 1 to 10 years).

The parents (18 mothers) were aged 43 to 60 years (*M* = 53.20, *SD* = 4.35). The deceased person was the child’s father (*n* = 10), brother (*n* = 4) or sister (*n* = 4), and the person had died by suicide (*n* = 13), accident (*n* = 2), manslaughter (*n* = 1), illness (*n* = 1) and undetermined (*n* = 1), on average 5 years previously (*M* = 5.31, *SD* = 2.89, range 1.5 to 10 years).

The clinicians (28 females, 6 males) were aged 26 to 71 years (*M* = 48.47, *SD* = 11.35). About one in four clinicians had five to nine years of experience (*n* = 8, 23.5%), 12 others (35.3%) had 10 to 19 years, and 14 (41.2%) had more than 20 years of experience.

#### 1.2.2. Data Collection and Analyses

Participants could choose between taking part in an individual interview by telephone or an in-person group interview [37,38,39,40,41]. We conducted 28 individual interviews, and 11 group interviews with 44 participants. Individual interviews lasted, on average, 46 min (range 19–76), and group interviews were an average of 77 min (range 40–102).

The interview guide was adaptable for individual and group interviews. It consisted of open-ended questions allowing for probes and follow-up questions. The lead questions addressed different aspects of the help (for example: “In your opinion, what help should be provided to a bereaved adolescent?”, “How should the help be provided?”, “How long after the loss?”, “What is the role of professional versus peer support?”, “What are the characteristics that make help helpful?”). Adopting an inductive approach, we analyzed the interview data through a codebook-based thematic analysis [42,43,44].

## 2. Materials and Methods

### 2.1. Survey

We created a short survey with five questions to assess the participants’ experiences of taking part in the individual or group interviews. Table 1 lists the survey questions. Participants were asked to answer the first four questions on a 5-point Likert-type scale (1. Not at all; 5. Absolutely). After each question, participants could write a free text comment. Question five was an open-ended question asking the participant if anything important to them was not discussed during the interview. The survey was anonymous, did not collect sociodemographic data, and could be filled out in less than five minutes.

### 2.2. Sampling

Participants who took part in an in-person group interview received a hard copy of the survey at the end of the interview, and/or by email, as requested. We emailed the survey to those who had taken part in a telephone interview. We asked all participants to return the survey within two weeks, and 85% (61 out of 72) did so. This included 85% (17 out of 20) of the adolescents, 67% (12 out of 18) of the parents, and 94% (32 out of 34) of the clinicians. This amounted to 96% (*n* = 42) of group interview participants and 68% (*n* = 19) of individual interview participants.

Of those who returned the survey, 54% (*n* = 33) provided at least one comment in the free text boxes of the four survey questions and the open-ended question. This included 41% (*n* = 7) of the adolescents, 58% (*n* = 7) of the parents, and 59% (*n* = 19) of the clinicians. There was no difference between the three groups (χ^2^ (2) = 1.589, *p* = 0.452). Additionally, an equal proportion of individual (53%, *n* = 10) and group participants (55%, *n* = 23) provided comments (χ^2^ (1) = 0.024, *p* = 0.877).

### 2.3. Analyses

We uploaded all data into SPSS version 26 [45]. We analyzed the quantitative data descriptively and results are presented as frequencies and percentages. Levels 1 and 2 of the 5-point Likert-type scale are considered as low, level 3 is moderate/medium, and levels 4 and 5 are high. We used the Kruskal–Wallis H test to test if there were differences between the three groups of participants. We used Kendall’s tau-b correlation coefficient (2-tailed) to investigate the correlations between the data derived from the four Likert-type questions.

As most comments provided in the free text boxes were short (a few words or a short sentence), we opted to summarize the qualitative data allowing for a quantitative and qualitative report of the findings [46]. The summary applied a deductive approach, based on the survey questions. Two researchers (KA and KK) summarized the data independently and compared their report; there were no discrepancies. The research team discussed the progress and results to maximize consistency throughout the study.

### 2.4. Ethical Approval

The Human Research Ethics Committee of The University of Melbourne approved the study (ID 1955213). All participants provided written informed consent.

## 3. Results

### 3.1. Quantitative Findings

#### 3.1.1. At the Time of Participating, Did You Feel Distressed When You Participated in the Focus Group/Interview?

**Figure 1:**
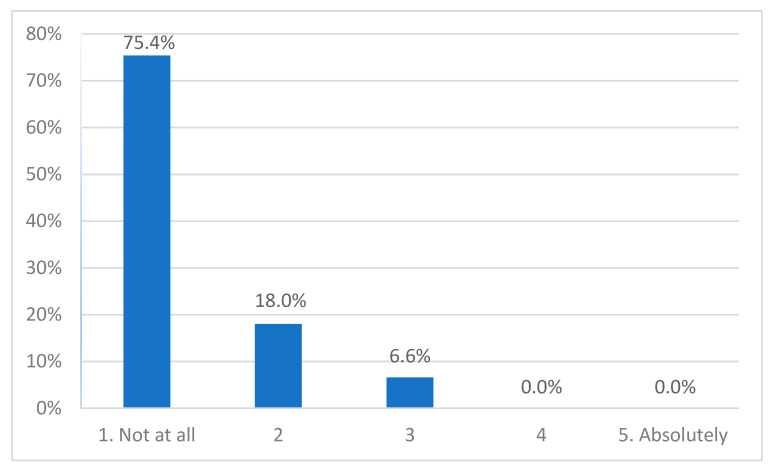
Experienced level of distress.

Most participants (75%, *n* = 46) reported that they had no or hardly any distress (18%, *n* = 11) while participating in the interview. Four participants (6.6%, three adolescents, one parent) reported moderate levels, and none reported high levels of distress (Figure 1). The Kruskal–Wallis test indicated a significant difference between the three groups (*H*(2) = 11.772, *p* = 0.003). Pairwise comparisons showed that adolescent participants scored higher than clinicians (*adj. p* = 0.002).

#### 3.1.2. Today, Do You Think That Participating Helped You in Anyway?

**Figure 2:**
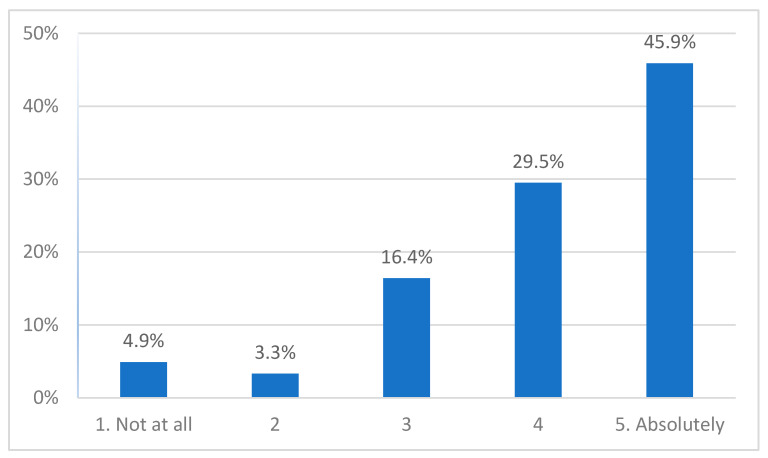
Perceived helpfulness.

About 75% (*n* = 46) of participants reported they felt that participation was helpful, 16% (*n* = 10) reported a medium level of perceived helpfulness, and 8% (2 adolescents, 1 parent, 2 clinicians) reported low levels (Figure 2). There was no difference between the three groups (*H*(2) = 0.210, *p* = 0.900).

#### 3.1.3. Today, Do You Feel That Participating Had Any Negative Effects for You?

**Figure 3:**
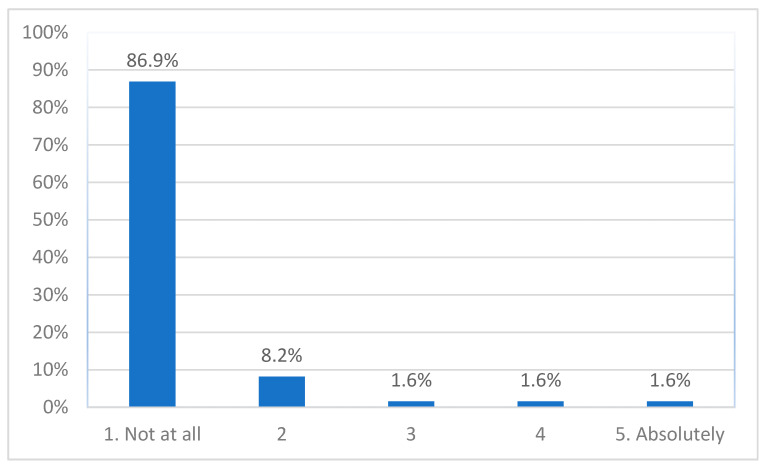
Experienced negative effects.

About 95% (*n* = 58) of participants reported having experienced no or hardly any negative effects of participating (Figure 3); 1.6% (1 adolescent) reported a moderate level, and 3.2% (1 adolescent, 1 parent) a high level of experienced negative effects. The Kruskal–Wallis test found a significant difference between the three groups (*H*(2) = 11.836, *p* = 0.003). Pairwise comparisons showed that adolescent participants scored higher than clinicians (*adj. p* = 0.002).

#### 3.1.4. Would You Recommend Participating in a Study Like This to Others?

**Figure 4:**
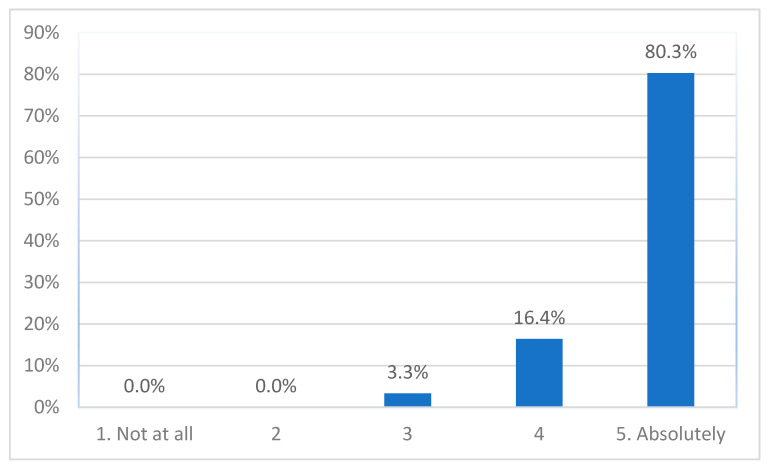
Recommending taking part to others.

Almost all participant (97%, *n*= 59) said they would absolutely or close to absolutely recommend participating in a study like this to others, with the other 3.3% (2 parents) being somewhat likely to recommend (Figure 4). There was no difference between the three groups (*H*(2) = 0.568, *p* = 0.753).

#### 3.1.5. Correlation Analysis

Table 2 presents the correlations, showing that there were significant associations between questions 1 and 3 (*p* = 0.007), and questions 2 and 4 (*p* = 0.026). This reveals that greater feelings of distress during participation were weakly related to more negative experienced effects of participation, and that greater perceived helpfulness of participation was weakly related to recommending participation to others.

### 3.2. Qualitative Findings

#### 3.2.1. At the Time of Participating, Did You Feel Distressed When You Participated in the Focus Group/Interview?

Six participants provided a comment: 2 adolescents, 1 parent, and 3 clinicians. Two adolescents reported having felt some anxiety but were otherwise fine, as stated by one: “Have anxiety but it was really okay”. One parent reported feeling “distress” after hearing stories of other participants, but the participant continued that this was experienced as a “helpful connection and catharsis”, which helped to reflect “how far” they “had come in their own grief”. Three clinicians reported having felt safe and supported while participating in the interview, as stated by this clinician: [It was] “Very supportive and informative”.

#### 3.2.2. Today, Do You Think That Participating Helped You in Anyway?

Twenty-two participants commented on this question: 5 adolescents, 5 parents, and 12 clinicians. Four adolescents and two parents experienced participating as being helpful for themselves, as stated by this adolescent: “It helped me open up and share my experience”. One adolescent and three parents referred to being able to use their experiences to help others in similar situations, for example, one parent wrote: “I appreciate being able to share to help those behind me”. Eight clinicians commented that participating in the interview was a learning experience, as stated by this clinician: “It’s always beneficial to talk about one’s practice and reflect on the strategies used in your daily work”. Three other clinicians experienced the interview also as a validation and encouragement, as said by this clinician: “It reminds practitioners to continue doing great work”. Still, one clinician commented on the interview as a “one-way conversation”.

#### 3.2.3. Today, Do You Feel That Participating Had Any Negative Effects for You?

Two participants noted a comment (1 adolescent, 1 parent). The adolescent stated that: “It brought up unpleasant memories but not overwhelming”. The parent reported having felt sad and having cried after the interview “particularly after listening to other participants, their loss of their loved ones”.

#### 3.2.4. Would You Recommend Participating in a Study Like This to Others?

Seven participants provided a comment (2 parents, 5 clinicians). The parents emphasized the importance of listening to others “across cultures and genders” to “identify evidence-based approaches that will work”. In addition, it may help us “learn how to best equip and cope with a suicide loss”. The clinicians would recommend participating because it “gives support and facilitates time to reflect, focus, and share ideas about a neglected cohort”. In addition, it can “increase education and awareness” for clinicians, and “support for young people”.

#### 3.2.5. Was There a Topic Very Important to You That You Thought We Should Discuss but Did Not?

Eighteen participants (4 adolescents, 3 parents, 11 clinicians) wrote a comment in the last free text box of the survey, although several comments appeared to be more about what participants thought was important regarding grief and bereavement, rather than about topics that were not addressed in the interviews. Three adolescents emphasized what they saw as crucial for helping bereaved adolescents. One wrote: “trust and knowing that there is good information and discussion out there”, and another highlighted the “need to let the younger teens know it’s not weak to speak and get help”. In addition, one parent elaborated on the “secrecy around suicide” and the tendency of blaming someone for a suicide, which may hinder bereaved adolescents. Subsequently, this parent argued for increased literacy around dealing with grief in society. Two clinicians pointed at cultural sensitivity in providing help, and financial impact of the loss, as important topics for further discussion.

One parent commented on the composition of the group in which she participated. Most participants in her group had lost a husband to suicide and only one participant had lost a child to suicide. Hence, the participant recommended that groups in future studies could be more balanced regarding types of relationship.

Twelve participants (1 adolescent, 2 parents, 9 clinicians) confirmed that everything important had been discussed in their interview, as exemplified by one adolescent who noted: “I believe we covered everything vital”, and participants expressed gratitude for having been involved, as stated by this clinician: “I’m sure there is more, but right now it feels good”.

## 4. Discussion

This study was a first to investigate the reactions of adolescents, parents, and clinicians regarding their participation in a study on adolescents bereaved by suicide and other traumatic death. Most participants reported that they experienced no distress while participating and no negative effects of participating. Rather, they found participation helpful for them and said they would definitely recommend participating in a study like this to others. These findings reflect results of bereavement studies with parents [47,48], siblings [49], and people bereaved by suicide [30,32], which reported that research participation was mostly a positive experience, with few negative experiences, and participants perceiving participation as being beneficial for themselves and others [30,32,47]. In addition, the correlation analysis showed that there is no contradiction between reporting distress or negative effects of participation and experiencing participation as helpful and recommending it to others. This finding is corroborated by suicide-related research with adolescents and trauma research with adults suggesting that emotional distress can be understood as an indicator of engagement in a data collection process rather than as an indicator of harm [50,51,52].

Although the overall levels of distress and negative effects reported in our study were low, adolescents reported the highest scores, and a few mentioned having experienced anxiety or unpleasant memories. This has also been reported with regard to adolescents participating in health-related studies [53]. As suggested above, these emotional reactions may be due to participants’ engagement with the research project [51], and a study by Hawton and colleagues [34] suggested that this may lead to them benefitting more from participating than adults. However, it may also point to the presence of emotional problems [53,54]. One study in a systematic review [53] examining children’s and adolescents’ reactions to participating in biomedical and health-related studies found that the presence of emotional problems in children was the only variable associated with short-lived negative research participation experiences [55]. Objective variables such as age, gender, methods of data collection, and topic/health condition examined in the studies, were not associated with participants’ appraisals of study participation [53,55]. Another study on pain also reported that age and level of pain did not affect responses regarding experiences of research participation [56]. However, as in our study, young participants still recommended participation to others [56]. Nonetheless, while only a few participants reported negative experiences, the phenomenon needs further investigation in bereaved adolescents, especially since both short-term deterioration and improvement in mood have been reported in adolescent mental health research [54].

Parents in our study were very positive about research participation. This is corroborated by other research with parents who participated in a study concerning the sudden and traumatic death of their children [27]. All parents in that study reported positive experiences and none regretted participating, despite 73% experiencing the interviews as (a little to very) painful [27]. As in our study, having the opportunity to share experiences and being able to help others contributed to a positive experience [27]. Still, one parent in our study reported being emotionally affected, although this parent still perceived participation as helpful. Follow-up data from adults participating in suicide research revealed that participants can be susceptible to short-term deteriorations in mood [57]. However, any negative effects of participation were confined to the days immediately following the study and this temporary deterioration in mood did not increase risk of suicidal thoughts [57].

As with research with bereaved adolescents, research with suicide bereaved adults indicated that objective factors such as gender of participants, their relationship to the deceased, the method of suicide, and time since loss appeared to have little effect on their experiences of participating in a research interview [58]. Moreover, research regarding mental health in adults showed that the presence of mental health problems did not differentiate participants with negative or positive experiences of research participation [59]. Hence, it may be that the presence of emotional problems has a stronger impact on the research experience of children and adolescents compared to adults, though further research is needed to ascertain such differences between children/adolescents and adults.

Clinicians in our study found research participation to be a learning experience. This is supported by findings from studies with clinicians who had lost a patient to suicide, who reported their research participation as a learning and therapeutic experience [60,61]. The finding is also in line with literature on substance abuse research participation [62]. Clinical research participants were more willing to use research findings in practice, especially those with favorable attitudes toward evidence-based practices and whose agencies supported professional growth. The combined findings of our study and others from the literature [60,62] suggest that research participation may reinforce clinicians’ willingness to use research findings in practice, thus contributing to quality of services.

Although our participants expressed gratitude for being involved in the research, a phenomenon also noted in the literature [63], our data suggest that from the three groups of participants, adolescents potentially experience the most distress and negative effects of participation in research interviews. Thus, researchers must inform potential participants, especially adolescents, about both the potential benefits and distress of research participation, and implement appropriate participant distress management protocols. As Parkes [64] pointed out, if a participant becomes distressed, “the needs of the respondent should take priority over the needs of the research” (p. 174). According to such protocols, researchers must provide support to a distressed interview participant according to the level of distress. This can range from allowing them to pause or withdraw from participation, providing emotional support to participants, providing or arranging referral to an appropriate support service (which may include a parent or guardian), or calling medical emergency services.

The literature indicates the important roles of narrating and sharing grief experiences in the processes of meaning-making and personal growth in those bereaved by suicide, which may also contribute to a positive experience as a study participant [10,58]. Interactions with a skilled and empathetic interviewer can also contribute to positive experiences for bereaved study participants [48,65]. While researchers must be sensitive to potential distress in participants, and provide emotional support if needed, they must also be aware of the potential methodological and ethical challenges when research and therapy blur [50,66]. For example, Biddle and colleagues [50] cautioned that participants may share information that they do not want to be used for the research, and researchers may not have the skills or capacity to deal with the distress or unintended shared information from the participant [50,64,66]. Hence, our findings and the broader literature imply that research interviews must be conducted by experienced and properly trained interviewers who can deal with participants’ emotions and make judgements about pausing or continuing an interview, or referring participants to external support [50,64,66]. Further research is needed to clarify the role of the researcher and their approach to participants regarding balancing data collection and being empathic and supportive for the research experience of bereaved participants [48,65,67,68].

To fully understand the study findings, it is important to note that the study involved participants who volunteered to share their experiences, and study participants may not be representative of the population from which they are recruited. In a bereavement study, Akard and colleagues [69] found that those who are motivated and have the capacity to participate tended to respond to the initial researchers’ invitations, and sending more than three invitations hardly increased the response rate [69]. Bereaved people who perceive research participation as too difficult may either decline participation or refuse passively by not responding [69,70]. These findings of the literature indicate the soundness of (potential) participants’ judgements about research participation [50] and indicate that participants make appropriate cost–benefit appraisals of their participation [52]. Research is needed to confirm these observations in adolescents bereaved by suicide, parents or other family members of bereaved adolescents, and clinicians. Nonetheless, researchers and research ethics committees may consider these when designing and assessing research studies in this field. In addition, participants in our study had experienced the bereavement at least six months before participating, which may also have contributed to participants reporting little distress.

### Limitations

The study involved a modest sample from a qualitative interview study. Despite the high response rate, the findings may not reflect the experiences of those who were invited to participate and chose not to. It is also not known whether findings apply to participants of other interview studies or studies utilizing other methods of data collection. Further, the survey did not include definitions of ‘distress’ or ‘negative effects’, and data were collected only at one point in time shortly after research participation. Future studies could include pre- and post-measures and longitudinal follow-up to assess any longer-term consequences.

## 5. Conclusions

The study clearly indicates that bereaved adolescents, parents and clinicians can safely participate in research interviews regarding their experiences of grief and help after suicide and other traumatic death. Participants reported that they experienced little distress and would recommend participation to others. To prevent and mitigate potential distress, training of research staff and implementation of appropriate participant distress protocols are imperative. Future studies could include longitudinal follow-up of participants.

## Figures and Tables

**Table 1:** Survey questions.

Questions
At the time of participating, did you feel distressed when you participated in the Focus Group/interview? Today, do you think that participating helped you in anyway? Today, do you feel that participating had any negative effects for you? Would you recommend participating in a study like this to others? Was there a topic very important to you that you thought we should discuss but did not? If yes, please describe.

**Table 2:** Correlations.

	1	2	3	4
1. At the time of participating, did you feel distressed when you participated in the Focus Group/interview?	1			
2. Today, do you think that participating helped you in anyway?	0.056	1		
3. Today, do you feel that participating had any negative effects for you?	0.335 **	−0.071	1	
4. Would you recommend participating in a study like this to others?	0.000	0.264 *	0.077	1

*. Correlation is significant at the 0.05 level (2-tailed). **. Correlation is significant at the 0.01 level (2-tailed).

## Data Availability

According to the ethics approval, data can only be accessed by the members of the research team.
